# Mapping epitopes on CRF01_AE viruses recognized by broadly neutralizing antibodies in sera from elite neutralizers from North America and Thailand

**DOI:** 10.1186/1742-4690-9-S2-P90

**Published:** 2012-09-13

**Authors:** SM O'Rourke, R Sutthent, KL Limoli, P Phung, GP Tatsuno, B To, KA Mesa, N Frigon, KW Higgins, T Wrin, PW Berman

**Affiliations:** 1 University of California, Santa Cruz, Santa Cruz, CA, USA; 2 Microbiology Department, Siriraj Hospital, Mahidol University, Bangkok, Thailand; 3 Monogram Biosciences, South San Francisco, CA, USA; 4 Global Solutions for Infectious Diseases, South San Francisco, CA, USA

## Background

The RV144 trial has rekindled interest in defining the epitopes responsible for the neutralization of CRF01_AE (clade E) viruses. In this study we used a novel method, swarm analysis, to map epitopes on Thai viruses recognized by broadly neutralizing (bN) antibodies in HIV+ sera. Swarm analysis relies on the swarm of closely related quasi-species that evolve in each HIV+ individual. In these studies we analyzed the bN activity in sera from elite neutralizers (ENs) against Thai viruses.

## Methods

Libraries of envelope genes were amplified from 30 Thai subjects who became infected with HIV in the VAX003 clinical trial. 12-24 clones from each individual were tested for sensitivity and resistance to neutralization by HIV+ EN sera obtained in Thailand and the USA. Pairs of neutralization sensitive and resistant viruses were identified from each individual, and the amino acids responsible for neutralization sensitivity localized by mutagenesis.

## Results

Novel mutations were identified that conferred neutralization sensitivity or resistance to antibodies from ENs. Unlike previous studies with clade B viruses, the mutations in clade E viruses that altered neutralization sensitivity and resistance did not appear to affect the CD4 binding site or gp41. The mutations identified were located in V1, V2, and V3 domains of gp120 and had no effect on neutralization by bN monoclonal antibodies such as VRC01, b12, PG9, PG16, and 2G12.

## Conclusion

Clade E viruses from Thailand can be neutralized by HIV+ sera from ENs infected with clade B or clade E viruses. The epitopes on clade E viruses recognized by bNAbs from ENs appear to be distinct from those defined with most of the bN monoclonal antibodies described to date. The results suggest that antibodies to the V1 domain as well as the V2 domain should be considered in the RV144 correlates analysis.

